# Mapping Quantitative Observer Metamerism of Displays

**DOI:** 10.3390/jimaging9100227

**Published:** 2023-10-19

**Authors:** Giorgio Trumpy, Casper Find Andersen, Ivar Farup, Omar Elezabi

**Affiliations:** Department of Computer Science, Norwegian University of Science and Technology, 2815 Gjøvik, Norway; casper.andersen@ntnu.no (C.F.A.); ivar.farup@ntnu.no (I.F.); omar.r.elezaby@gmail.com (O.E.)

**Keywords:** display technology, color reproduction, visual perception, observer variability

## Abstract

Observer metamerism (OM) is the name given to the variability between the color matches that individual observers consider accurate. The standard color imaging approach, which uses color-matching functions of a single representative observer, does not accurately represent every individual observer’s perceptual properties. This paper investigates OM in color displays and proposes a quantitative assessment of the OM distribution across the chromaticity diagram. An OM metric is calculated from a database of individual LMS cone fundamentals and the spectral power distributions of the display’s primaries. Additionally, a visualization method is suggested to map the distribution of OM across the display’s color gamut. Through numerical assessment of OM using two distinct publicly available sets of individual observers’ functions, the influence of the selected dataset on the intensity and distribution of OM has been underscored. The case study of digital cinema has been investigated, specifically the transition from xenon-arc to laser projectors. The resulting heatmaps represent the “topography” of OM for both types of projectors. The paper also presents color difference values, showing that achromatic highlights could be particularly prone to disagreements between observers in laser-based cinema theaters. Overall, this study provides valuable resources for display manufacturers and researchers, offering insights into observer metamerism and facilitating the development of improved display technologies.

## 1. Introduction

A human observer is characterized by three *cone fundamentals* (LMS) that quantitatively describe how the photoreceptors respond to different wavelengths of light entering the eye, which in turn influences our perception of color [1]. *Metamerism* is a phenomenon in color perception where two stimuli with different spectral power distributions (SPDs) produce the same cone activation in an observer. Thus, they appear to have the same color in equivalent contexts. Thanks to metamerism, display technology can accurately reproduce color by producing *metamers* (i.e., color matches) using only three RGB primaries.

In the standard terminology [2,3], while *metamerism* indicates a color match in a single observation of a pair of stimuli, the specifications of metamerism “*illuminant metamerism*” and “*observer metamerism*” refer to two separate observations of a stimuli pair, and in this case, the term *metamerism* indicates a difference between the observations (not a match). *Observer metamerism* (OM) is the name given to the situation in which two stimuli with different SPDs have the same color for one observer and have different colors for another observer. OM is the evidence for the variability between the metamers that individual observers consider accurate. This variability is known to be significant [4,5].

In standard imaging technology, color reproduction is carried out referring to a single representative observer with defined *color-matching functions* (CMFs), which are generally the CIE 1931 XYZ 2° Standard Observer (indicated as *2-CMF1931*) [6]. Since the standard observer does not exactly represent the perceptual properties of every individual observer, there might be observers that do not consider accurate the reproduction of natural colors with an RGB display that has been precisely characterized [7].

The present work introduces a method to quantitatively evaluate the OM of a display for all its reproducible colors (*color gamut*). An OM metric is directly calculated from the SPD of the display’s primaries, considering a dataset of individual LMSs. Since some colors are more susceptible to disagreement between observers than others, a visualization method is proposed that maps how the possibility of disagreement—expressed by the OM metric—varies across the display’s gamut.

**Figure 1:**
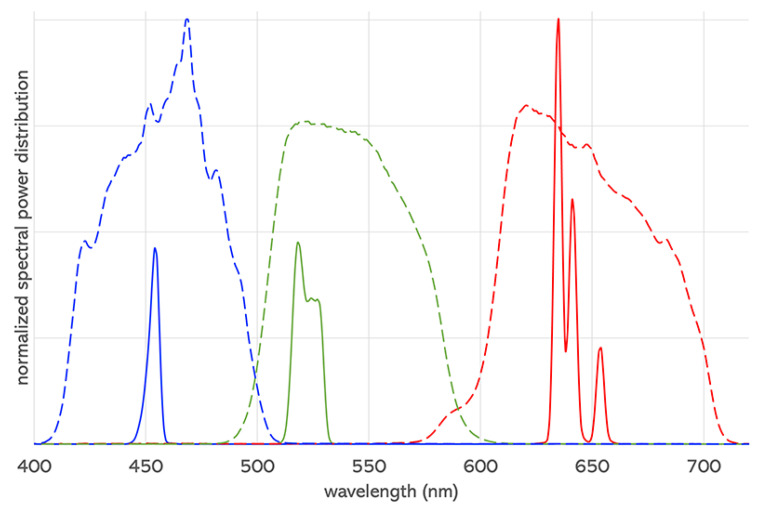
Spectral power distributions of the RGB primaries of a laser (solid lines) and a xenon-arc (dashed lines) DLP cinema projector, normalized to the peak of their most powerful primary.

A numerical and visual representation of the effect of OM can support the design of future display devices, facilitating the understanding and control of the disagreement between observers. The evaluation and visualization are presented with a case study related to digital cinema. Cinema display technology has undergone significant advancements over the years [8]. Currently, we are seeing the transition from xenon-arc lamps to lasers in Digital Light Processing (DLP) projectors. Laser-based projectors have a wider color gamut thanks to the very narrow spectral emission of this type of light source (see Figure 1). However, while the extended gamut can deliver enhanced visual experiences for moviegoers, the rapid changes in emitted light in a small interval of wavelengths cause a higher observer metamerism [9]. The repercussions of OM in the transition between xenon-arc to laser in cinema theaters have created concern [10]; for this reason, we decided to use this example to present our method.

## 2. Related Work

Research has been conducted to investigate the phenomena of observer metamerism and its effect on different technologies and the degree of observer metamerism in existing devices and different solutions to overcome this problem. The solutions that have been proposed to mitigate the effect of OM include displays with ideal sets of three primaries [9,11] as well as multi-primary display systems, which cover a wide color gamut while containing the observer metamerism [12]. Personalized color management systems have also been proposed to minimize OM based on matrix corrections [13], new observer categories [14], or new LMS cone fundamentals [15]. These solutions are potentially successful for individual displays, such as personal computer monitors and head-mounted displays. However, they are not suitable for cinema or other settings in which visual content is distributed in public spaces.

Quantitative evaluations of OM have been attempted for quite some time. A CIE observer metamerism index was introduced as early as at the end of the 1980s [16] that considered four deviation functions characterizing the variations in color-matching functions of color-normal observers. It was later highlighted that the inter-observer variability in experimental data is much larger than what is predicted by the CIE model [17].

Most recent studies evaluate the OM of displays using datasets of CMFs that are inferred through mathematical models starting from experimental data obtained from a few broadband stimuli [18,19]. Based on this type of CMFs database, metrics have been developed to characterize the OM of displays with a single average value of a set of colors [20] and displaying distributions of OM over the color gamut [21]. Another quantitative approach to assess the effect of OM with a single value is based on the principal angles between the vector subspaces spanned by the display primaries multiplied with the *2-CMF1931* and a dataset of individual observers [22,23].

Arguably, conducting an OM assessment using entirely measured individual CMFs may yield more reliable results.

## 3. Materials and Methods

The current study introduces an impactful visualization that not only quantifies the OM of displays, but it also facilitates the identification of areas within their color gamut that are more likely to result in observer disagreement. The OM visualization is evaluated using two distinct datasets of individual observers.

### 3.1. The LMS Approach

**Figure 2:**
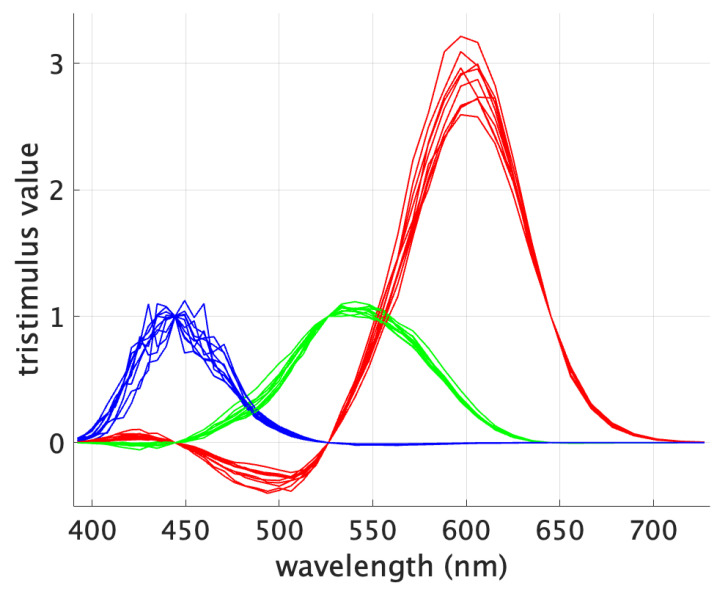
Stiles and Burch (1955) 2-deg individual color-matching functions for red, green and blue primaries.

Color-matching experiments have been the most practical and used way to measure color perception. Figure 2 shows the CMFs of a set of 10 individuals from the experiments conducted by Stiles and Burch in 1955 [24]. The tristimulus values correspond to monochromatic primaries at 444.4, 526.3 and 645.2 nm [25].

The data of the 10 observers converge at the wavelengths corresponding to the primaries used during the color-matching experiments. In fact, for those monochromatic stimuli, an agreement is reached by definition between all the observers since each primary will be best matched by itself despite the variability in the observers’ color perception. As a result, at the primaries’ wavelengths the tristimulus values are [0 0 1], [0 1 0] and [1 0 0].

To quantitatively assess OM, an approach to calculating differences in color perception is necessary. The colorimetric difference between stimuli that result in the same tristimulus values considering different individual CMFs can be assessed by a standard observer—acting as a “referee”—using established color difference equations. However, due to the crossings mentioned above (see Figure 2), if a display with narrowband RGB primaries—such as a laser-based projector—is assessed for OM, and the display’s primaries match the primaries of the individual CMFs, small color differences between observers would be obtained indicating a low OM. This would create an erroneous bias and a faulty OM assessment.

Some authors have formulated observer metamerism metrics using datasets of color-matching functions with varying primaries [20,21,26], assuming that the resulting colorimetric differences are indicative of the OM magnitude despite the inconsistent primaries. In these approaches, the XYZ values of different observers are compared directly. This is problematic for at least two reasons. First, when two observers have different CMFs, their tristimulus spaces are different subspaces of the space of physical spectra. Second, when the individual CMFs are mapped to XYZ-like curves by optimizing the linear transform, one ends up with CMFs that relate to different sets of primaries. It is by no means obvious that color difference equations developed for the standard observer will provide reasonable results in this setting.

In the present work, the OM of displays is quantitatively assessed considering the stimuli that produce the *same cone response* in different observers. The OM metric is derived from the color difference between these stimuli as judged by a standard observer. Quantitative maps that depict the magnitude of OM across the display’s gamut are calculated from two elements: (I) the SPD of the display’s primaries, and (II) a dataset of LMSs corresponding to a set of individual observers.

### 3.2. Measurement of Displays’ Primaries

The present work analyzes the case of two cinema DLP projectors: a xenon-arc projector (Barco DP2K-20C) and a laser projector (Christie CP4320-RGB). The spectral radiance of their primaries have been measured with a spectroradiometer (Konica Minolta CS-2000A) pointing at the center of the screen from a central seat of the theater and using the MCGD (Measured Color Gamut Data) panel of the projector’s software [27]. The measured spectral emissions are plotted in Figure 1.

### 3.3. Datasets of Individual Cone Fundamentals

**Figure 3:**
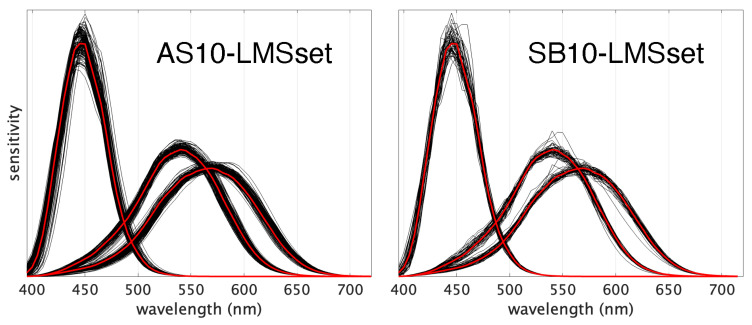
The two 10-deg datasets of cone fundamentals used in the calculations (black lines): AS10-LMSset constituted of 151 individuals (**left**) and SB10-LMSset (**right**) constituted of 51 individuals. In both plots, the Stockman and Sharpe (2000) 10-deg cone fundamentals are reported (red lines). All the functions in the two plots are normalized to the same area.

To evaluate the impact of the chosen dataset of individual observers on the distribution of the OM metric across the color gamut, we conducted tests deriving individual LMSs from two publicly available datasets of individual CMFs [28,29]. In addition to originating from distinct sets of individuals and being published decades apart, these two datasets are also the result of markedly different experimental approaches. The “AS10-LMSset” functions (Section 3.3.1) are inferred through broadband color matching, while the “SB10-LMSset” functions (Section 3.3.2) are derived from experiments involving maximum saturation color matching. As Figure 3 shows, the bundles of curves exhibit noticeably greater variability for “AS10-LMSset” compared to “SB10-LMSset”. This disparity noticeably influences the OM visualization, as detailed in the forthcoming sections.

#### 3.3.1. Calculated Cone Fundamentals from Inferred CMFs—AS10-LMSset

The first set of individual cone fundamentals is constituted of the 151 LMSs published by Asano [28]. Here, the LMSs are inferred optimizing eight physiological parameters, which are estimated from broadband color matching using an RGB electronic display to match reflected SPDs from illuminated surfaces. The 151×3 cone sensitivities are sampled in the 390–780 nanometer range with a 5 nm step (Figure 3-left) and normalized to the same sum value, so all observers refer to the same white-point (equal-energy, i.e., CIE-E).

#### 3.3.2. Estimated Cone Fundamentals from Measured CMFs—SB10-LMSset

The second set of individual cone fundamentals has been estimated from the Stiles and Burch maximum saturation color-matching experiment data that were conducted with 51 individual observers, considering a 10° arc around the fovea [24]. The sensitivities are sampled in the 395–715 nanometer range with a 5 nm step, corresponding to 65 samples (Figure 3-right) and are found with a slightly modified version of the method of estimating individual LMSs from their according CMFs, which is presented in [30]. The aim of the method is to determine the actual matrix pertaining to an individual that will linearly transform its color-matching functions to its corresponding individual cone fundamentals. The *assumption* in this method is that an *i*th individual CMF ${\underline{c}}_{i}$ is linearly related to a spectrally filtered set of pre-defined target absorptances [31] denoted ${\underline{\alpha }}_{t}\left(\lambda \right)$ so that:

(1) $$f_{i}\left(\lambda \right){\underline{\alpha }}_{t}\left(\lambda \right)={\underline{c}}_{i}\left(\lambda \right)M_{i}$$

where $f_{i}\left(\lambda \right)$ defines a transmissive filter, denoted *prefilter*, and $M_{i}$ is a 3×3 matrix. In theory, the prefilter could be physically represented by a color filter positioned along the path of light before it reaches the cornea. This filter would thereby modify the spectral properties of the light before its interaction with the retina. The spectral transmission of the prefilter is within the present definition, either fully or partly containing the lenticular and macular filtration. When using ${\underline{l}}_{i}\left(\lambda \right)$ to represent the *i*th cone fundamentals, then:

(2) $${\underline{l}}_{i}\left(\lambda \right)={\underline{c}}_{i}\left(\lambda \right)M_{i}$$

or equivalently:

(3) $${\underline{l}}_{i}\left(\lambda \right)=f_{i}\left(\lambda \right){\underline{\alpha }}_{t}\left(\lambda \right)$$

Discounting noise from factors such as experimental inaccuracy or perceptual thresholds, if the target absorptances are in accordance with the real absorptances denoted ${\underline{\alpha }}_{i}\left(\lambda \right)$ (i.e., if ${\underline{\alpha }}_{t}\left(\lambda \right)={\underline{\alpha }}_{i}\left(\lambda \right)$), then the assumption in Equation (1) along with Equations (2) and (3) are correct and the prefilter $f_{i}\left(\lambda \right)$ will consist of the combined macular and lenticular transmission. However, if this is not the case due to genotypical variations between the target and the real absorptances, then the assumption is likely to only be approximately correct. To generalize the assumption in Equation (1) to any ${\underline{c}}_{i}$ and a chosen, fixed ${\underline{\alpha }}_{t}$, we define a linear least squares optimization scheme in which estimates of a prefilter ${\hat{f}}_{i}\left(\lambda \right)$ and a matrix ${\hat{M}}_{i}$ are found that minimize the error $\Delta_{i}$ so that:

(4) $$\Delta_{i}=\min_{{\hat{M}}_{i},{\hat{f}}_{i}\left(\lambda \right)}{\left\|{\hat{f}}_{i}\left(\lambda \right){\underline{\alpha }}_{t}\left(\lambda \right)-{\underline{c}}_{i}\left(\lambda \right){\hat{M}}_{i}\right\|}^{2}$$

Upon discretization, Equation (4) is solved in a convergent, iterative, alternating scheme of coupled minimization, which enables a separation of the estimations of ${\hat{f}}_{i}\left(\lambda \right)$ and ${\hat{M}}_{i}$ until convergence [30]. It is important to notice that metameric matches are invariant to linear transformations, and therefore the OM is theoretically contained in ${\hat{f}}_{i}\left(\lambda \right)$. It is also important to acknowledge that ${\hat{f}}_{i}\left(\lambda \right)$ generally does not have a strict physiological interpretation; instead, it contains values that simply result from the optimization process.

Following Equation (2), the main results regarding the estimated individual cone fundamental functions ${\hat{\underline{l}}}_{i}\left(\lambda \right)={\underline{c}}_{i}\left(\lambda \right){\hat{M}}_{i}$ are only qualitatively discussed, but it is found that they are likely to be close to the, albeit unknown, real cone fundamentals ${\underline{l}}_{i}$ when allowing for estimation inaccuracy originating from the sampling frequency of the color-matching functions and instrumental and/or perceptual noise. The estimated individual prefilters contain the macular and lenticular transmissions, and are further believed to cancel the main part of the $\Delta_{i}$ error in the optimization. It is, thus, further concluded from the presented results and discussion that the estimated matrix is close to the actual individual matrix.

In [32], an optimization method is employed to estimate the spectral transmission function of a physically feasible colored filter to install in front of a camera with three spectral sensitivities (i.e., RGB) to yield camera responses closer to human responses to incident spectral stimuli. The method estimates the prefilter in accordance with Equation (4) when the CMFs ${\underline{c}}_{i}$ are replaced by a XYZ standard observer (e.g., 2-CMF1931) and the absorptances ${\underline{\alpha }}_{t}$ with the sensor’s spectral sensitivities. The premise of the method is that, given the variations introduced in practical color matching and noise in camera captures, the prefiltered (i.e., color filtered) and matrixed camera sensor functions (i.e., estimated XYZ functions) are close enough to XYZ in a colorimetric sense to be useful as a replacement for XYZ. Here, it is found that the perceptual error resulting from the differences between standard observer responses and the according responses from color-filtered camera sensors are on a par with other sources of noise, such as the camera capture and rendition process.

Since in typical digital cameras, the green sensitivity is shifted far away from the red sensitivity towards the blue sensitivity—as opposed to the cone fundamentals—the metamerism mismatch caused by differences between sensors and observers is likely to be larger than the OM. Therefore, we expect that a perceptual evaluation of the estimates found by Equation (4) would be even more favorable. In [33], (work in progress) an expansion of the method will be presented, in which a quantification of the accuracy of the estimated individual cone fundamentals based on the optimization method in (1) is included.

In the present paper, target cone fundamentals ${\underline{l}}_{t}$ are predetermined instead of ${\underline{\alpha }}_{t}$, as is the case in [30]. Defining $f_{t}\left(\lambda \right)$ to be the combined lenticular and macular transmission pertaining to the target, then:

(5) $${\underline{l}}_{t}\left(\lambda \right)=f_{t}\left(\lambda \right){\underline{\alpha }}_{t}\left(\lambda \right)$$

Inserting Equation (5) into (1) and letting $f_{i}^{★}=f_{i}\left(\lambda \right)f_{t}^{-1}\left(\lambda \right)$, then:

(6) $$f_{i}^{★}\left(\lambda \right){\underline{l}}_{t}\left(\lambda \right)={\underline{c}}_{i}\left(\lambda \right)M_{i}$$

which leads to a modified optimization equivalent to Equation (4):

(7) $$\Delta_{i}^{★}=\min_{{\hat{M}}_{i},{\hat{f}}_{i}^{★}\left(\lambda \right)}{\left\|{\hat{f}}_{i}^{★}\left(\lambda \right){\underline{l}}_{t}\left(\lambda \right)-{\underline{c}}_{i}\left(\lambda \right){\hat{M}}_{i}\right\|}^{2}$$

The continuous functions of wavelength λ in Equation (7) are in practice given in discrete values. So, to provide a solution to Equation (7), the optimization is converted to a discrete formulation. Upon discretization of Equation (7), in which ${\underline{c}}_{i}\left(\lambda \right)$ is replaced by $C_{i}$ and ${\underline{l}}_{t}\left(\lambda \right)$ is replaced by $L_{t}$, each of which are N×3 matrices, when defining *N* as the number of discrete wavelength steps within the interval of visible wavelengths, e.g., [390,395,…,730] nanometers, the estimated prefilter is replaced by a N×N diagonal matrix ${\hat{D}}_{i}^{★}$ where the diagonal elements are a N×1 dimensional vector of discrete values pertaining to ${\hat{f}}_{i}^{★}\left(\lambda \right)$, so that:

(8) $$\Delta_{i}^{★}=\min_{{\hat{D}}_{i}^{★},{\hat{M}}_{i}}{\left\|{\hat{D}}_{i}^{★}L_{t}-C_{i}{\hat{M}}_{i}\right\|}^{2}$$

The final goal is to estimate individual cone fundamentals ${\hat{L}}_{i}$ functions from their according color-matching functions $C_{i}$. The smaller $\Delta_{i}^{★}$ is, the more the estimates are believed to be true. Equation (8) will lead to:

(9) $${\hat{L}}_{i}=C_{i}{\hat{M}}_{i}$$

or equivalently, allowing for the size of $\Delta_{i}^{★}$:

(10) $${\hat{L}}_{i}={\hat{D}}_{i}^{★}L_{t}$$

Let the $C_{i}$, i∈{1,⋯,51} be the CMFs of the Stiles and Burch experiments [29] and $L_{t}$ be the Stockman and Sharpe (2000) 10-deg cone fundamentals [34] (indicated as *10-LMS2000*). The resulting dataset of individual observers represents a fundamental element of the present paper and is constituted by the cone fundamentals ${\hat{L}}_{i}$, i∈{1,⋯,51}. All 51×3 arrays representing the cone sensitivities are normalized to the same sum value, so all observers refer to the same white-point (equal-energy—CIE-E). The resulting dataset is referred to as *SB10-LMSset*.

### 3.4. Observer Metamers

It is established that two stimuli that produce the same cone activation in an observer are metamers, i.e., they appear to have the same color. A more debatable question is whether two different observers, each receiving a different stimulus that produces in them the same cone activation, see the same color. However, it can be assumed that the color difference between these two stimuli that evoke the same physiological response is a good assessment of the difference in color perception between the two individual observers.

Given an arbitrary starting stimulus generated by a color display characterized by its 3×1 RGB values, r, we want to find the corresponding stimuli that give the same cone response for each individual observer of the LMS dataset as the cone response of the standard observer 10-LMS2000 produced by the starting stimulus. If the spectral power distributions of the primaries of the display device are represented by the N×3 matrix P, the spectral power distribution of the stimulus is s=Pr. The cone responses of the 10-LMS2000 standard observer with cone fundamentals represented by the N×3 matrix L are $l=L^{T}\mathrm{Pr}$.

In order for the individual observer with cone fundamentals $L_{i}$, i∈{1,…,Q}, to have the same cone responses as the standard observer, the RGB triplets have to be adjusted for the individual into $r_{i}$ such that they produce the same cone response,

(11) $$l=L^{T}\mathrm{Pr}=L_{i}^{T}{\mathrm{Pr}}_{i}\;\Rightarrow \;r_{i}={\left(L_{i}^{T}P\right)}^{-1}L^{T}Pr$$

Thus, the Q metamers corresponding to RGB stimulus r are

(12) $$s_{i}={\mathrm{Pr}}_{i}=P{\left(L_{i}^{T}P\right)}^{-1}L^{T}Pr$$

**Figure 4:**
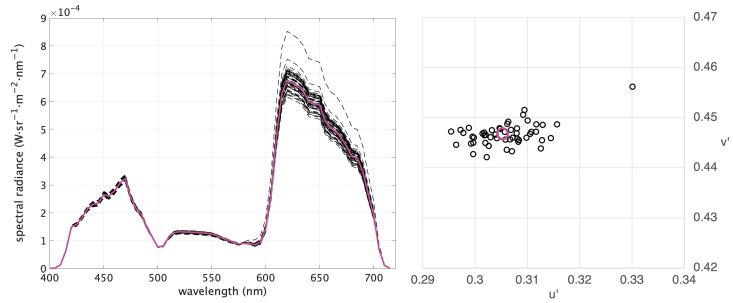
(**Left**): A set of observer metamers produced with a xenon-arc DLP projector for the 10-LMS2000 standard observer (solid magenta line) and the 51 individual observers of SB10-LMSset (black dashed lines). (**Right**): Corresponding chromaticity values in the CIE 1976 UCS plot, constituting an OM-cloud (standard observer in magenta and individual observers in black).

The chromaticity values corresponding to the Q+1 stimuli (the Q observer metamers $s_{i}$ plus the starting stimulus s) are calculated considering *2-CMF1931* and plotted in the CIE 1976 UCS diagram [35]. The resulting scatter plot is called the **Observer Metamerism cloud** (OM-cloud). Figure 4 illustrates a representative example of a magenta stimulus produced with the xenon-arc DLP projector and the 51 observer metamers calculated with SB10-LMSset. The spectra are reported on the left of Figure 4 and the scatter plot on the right is the corresponding OM-cloud.

**Figure 5:**
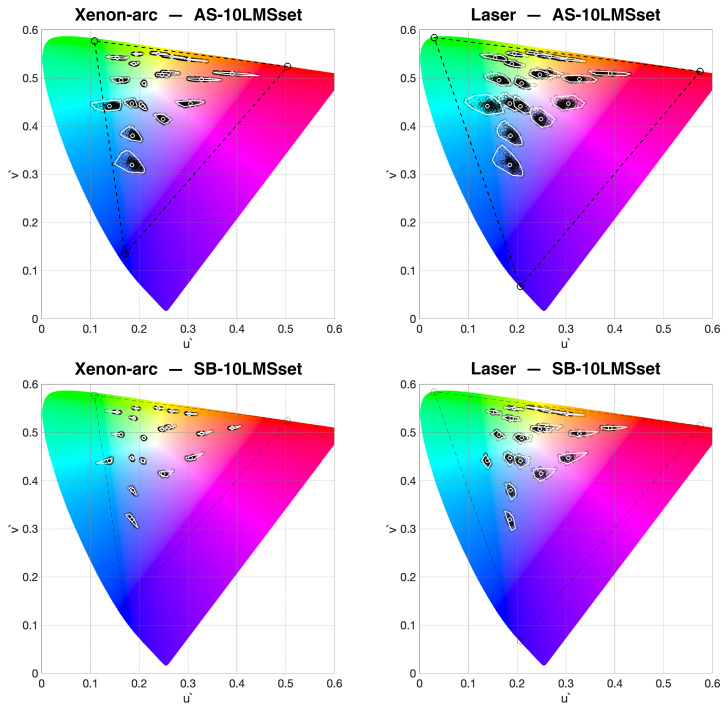
OM-clouds for xenon-arc (**left**) and laser (**right**) DLP projectors, whose primaries are reported in Figure 1. The white circles in the center of the clouds correspond to 10-LMS2000 and have the same positions in the two plots. The surrounding black circles correspond to the individual observers. The convex hull is marked in white for all clouds. The values are computed using AS10-LMSset (**top**) and SB10-LMSset (**bottom**).

Given a display device, an OM-cloud can be defined for any color, so the level of OM can be assessed for different regions of the display’s gamut. Figure 5 shows the OM-clouds for a set of 24 chromaticities corresponding to the Macbeth ColorChecker [36] (the six patches of the grayscale overlap around [0.209 0.488]). The OM-clouds corresponding to the same starting colors are computed for the two projectors and the two databases. By comparing the left diagrams in Figure 5 with the corresponding right ones, one can observe that—as expected—the OM-clouds of the laser projector are bigger than those of the xenon-arc projector due to the narrower spectral emission of the laser primaries (see Figure 1). By comparing the top diagrams in Figure 5 with the corresponding bottom ones, one can observe that the OM-clouds are significantly bigger if AS10-LMSset is used in the calculations compared to SB10-LMSset.

### 3.5. Observer Metamerism Index

The distance in the CIE 1976 UCS diagram between observer metamers is considered to be representative of the perceptual difference between two observers.

Assuming that the LMS datasets are actually representative of the variability in the cone fundamentals between different individuals, there is an equal probability for any observer to be accurately described by one of the LMS functions in the dataset. Therefore, the OM can be quantified by the mean distance between all combinations of two elements of the OM-cloud (Q×(Q−1)/2 combinations), which we indicate with $\bar{D}$. We define the **Observer Metamerism Index** (OM-index) with the following simple equation:

(13) $$\mathrm{OM}-\mathrm{index}=100\times \bar{D}$$

This metric is not only determined by the external size of the OM-cloud (e.g., the area of the convex hull) but it also considers its internal distribution.

## 4. Results

**Figure 6:**
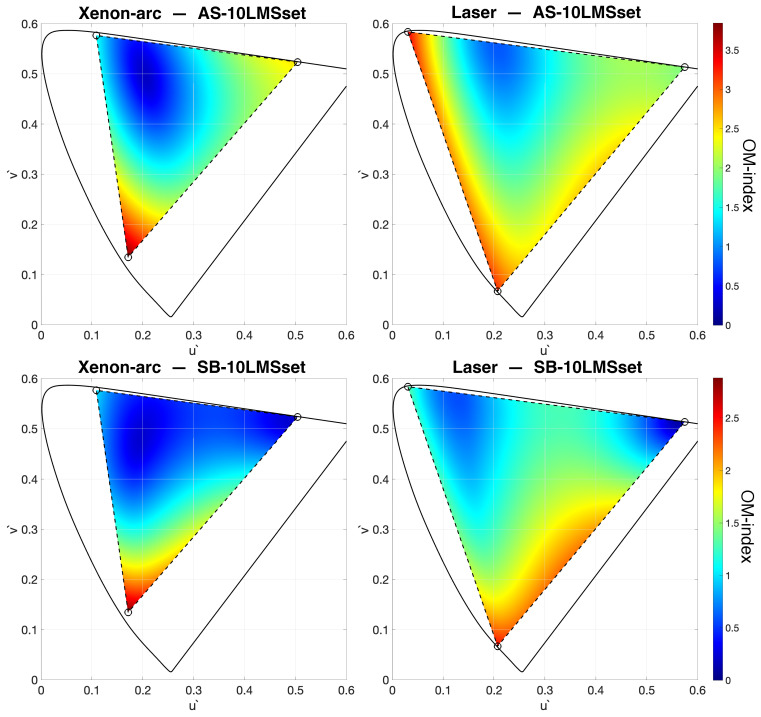
OM-index displayed as heatmaps for the whole gamut of the analyzed displays considering the two different datasets. Xenon-arc on the left and laser on the right, AS10-LMSset at the top and SB10-LMSset at the bottom.

For a given display, an OM-index can be calculated for any RGB triplet, so it is possible to quantitatively map the observer metamerism across the display’s gamut. The heatmaps in Figure 6 display the “topography” of the Observer Metamerism, expressed by the OM-index for the xenon-arc (diagrams on the left) and the laser projector (diagrams on the right). The values are computed using AS10-LMSset (diagrams along the top) and SB10-LMSset (diagrams along the bottom). It was decided to adjust the full-scale according to the maximum value within a specific dataset (3.83 for the top heatmaps and 2.87 for the bottom ones). This decision was made with practical application of the OM visualization method in mind, wherein only one dataset will be considered. By utilizing the full dynamic range of the heat palette, the effectiveness of the visualization process is optimized.

It is worth noting that the chromaticity values of observer metamers remain consistent regardless of the selected luminance value Y. Consequently, the heatmaps in Figure 6 are not influenced by changes in Y. This phenomenon can be attributed to the fact that we are considering the observation of individual stimuli, where luminance adaptation effectively normalizes the intensity. This becomes particularly relevant in the context of projectors, where the actual luminance levels are contingent upon the screen’s image size.

Not surprisingly, the xenon-arc projector has a lower average OM “temperature” than its laser counterpart (Table 1). However, the OM peak value of the xenon-arc projector, which is located at the blue primary, is higher than the peak value of the laser projector. The comparison between the top and bottom heatmaps in Figure 6 indicates two important things: (1) the choice of the dataset determines a significant difference in the overall level of disagreement between observers (note that the full scale of the OM-index’s color bars are different), and (2) the choice of the dataset changes the distribution of the intensities producing consistent discrepancies in the results. For example, when examining the saturated greens produced by the laser projector (top-left corner of the gamut’s triangle), AS10-LMSset diagnoses a high OM, whereas SB10-LMSset indicates a low OM for the same stimulus. Similarly, a discrepancy is seen for saturated reds produced with the xenon-arc projector (right corner of the gamut’s triangle) that exhibit rather high OM if AS10-LMSset is considered, while the same stimulus results in the lowest range of OM values if SB10-LMSset is considered.

**Figure 7:**
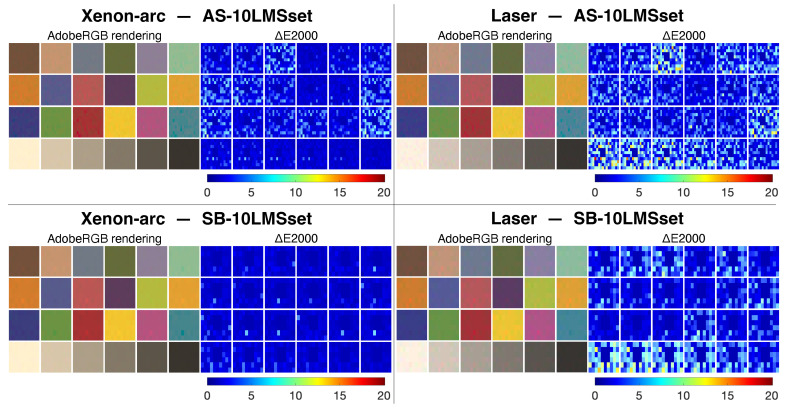
The four ‘OM-ColorCheckers’ visualizing the color differences between the observer metamers as Adobe RGB color renderings and as ΔE2000 heatmaps, considering the two displays and the two LMS datasets. Xenon-arc on the left and laser on the right, AS10-LMSset at the top and SB10-LMSset at the bottom.

To provide an effective visual representation of the color differences between the observer metamers, the color values of the Macbeth ColorChecker have been selected [36]. Considering the two projectors and the two LMS datasets, four sets of observer metamers have been calculated for each patch of the ColorChecker as described in Section 3.4. Each square patch is subdivided into Q+1 sectors (152 for AS10-LMSset and 52 for SB10-LMSset). For all 24×4 patches, a group of 3×3 sectors inside each patch has been merged into a single sector. This latter bigger sector is assigned to the 10-LMS2000 standard observer, and the other Q sectors are assigned to the individual observers of the dataset. Color values have been calculated considering *2-CMF1931*, and the four “*OM-ColorCheckers*” calculated in Adobe RGB (1998) [37] are reported in Figure 7 (left image of each pair). These visual renderings have to be considered just “desktop representation” of the actual colors produced by the DLP projectors.

The CIELAB values of the observer metamers have been calculated considering the projector’s native white-point. Color difference values were determined using the ΔE2000 formula [38] using 10-LMS2000 as the reference (i.e., the central bigger sector inside each patch). The color differences are shown in Figure 7 (right image of each pair) as heatmaps following the same arrangement of the color image.

Irrespective of the dataset selected, the ΔE2000 values are particularly high in the lighter patches of the grayscale for the laser projector, suggesting that achromatic highlights could be particularly prone to disagreements between observers in laser-based cinema theaters.

## 5. Discussion

This paper has explored the concept of observer metamerism in displays and has provided an effective visualization of its quantitative attributes. The novel approach confidently posits that the color difference between two stimuli that evoke the same physiological response serves as an assessment of the disparity in color perception between the two individual observers. The OM-clouds (Figure 5) and the distribution of the OM-index (Figure 6) represent valuable resources for display manufacturers, researchers, and practitioners working in color imaging. It has also come to light that selecting different available datasets of individual observers’ cone fundamentals leads to significantly distinct intensities and distributions of OM values across the display’s color gamut. This observation serves as a cautionary reminder about the crucial importance of establishing robust fundamentals for OM evaluation. The quantitative framework developed in this study has emphasized that achromatic highlights may be especially susceptible to disagreements between observers in laser-based cinema theaters.

Future work should include the verification of the findings with psychophysical experiments, confirming that the OM-index statistically well correlates with disagreements between observers in cross-media color-matching experiments.

## Figures and Tables

**Table 1:** Average and maximum OM-indices corresponding to the diagrams in Figure 6.

OM-Index	Xenon-Arc		Laser	
	avg	max	avg	max
AS10-LMSset	1.46	3.83	1.94	3.38
SB10-LMSset	0.86	2.87	1.31	2.50

## Data Availability

Data supporting the reported results are all contained within the paper.

## Abbreviations

The following abbreviations are used in this manuscript:

CMF	Color-Matching Functions (one set of three functions)
LMS	Cone Fundamentals (Long, Medium and Short)
OM	Observer Metamerism
SPD	Spectral Power Distribution
